# *Eutrema giganteum* (Brassicaceae), a new species from Sichuan, southwest China

**DOI:** 10.3897/phytokeys.82.12329

**Published:** 2017-06-29

**Authors:** Guoqian Hao, Changbing Zhang, Ihsan A. AL-Shehbaz, Xinyi Guo, Hao Bi, Junyin Wang, Jianquan Liu

**Affiliations:** 1 Biodiversity Institute of Mount Emei, Mount Emei Scenic Area Management Committee, Leshan 614000, P. R. China; 2 Sichuan Academy of Grassland Sciences, Chengdu 610065, P. R. China; 3 Missouri Botanical Garden, P.O. Box 299, St. Louis, MO 63166-0299, U.S.A.; 4 MOE Key Laboratory for Bio-Resources and Eco-Environment, College of Life Science, Sichuan University, Chengdu 610065, P. R. China

**Keywords:** Brassicaceae, Cruciferae, *Eutrema giganteum*, new species, *Eutrema yunnanense*, molecular phylogeny

## Abstract

*Eutrema giganteum* (Brassicaceae), a new species from Hengduan Mountains in Sichuan Province, southwest China, is described, and its relationships to the closely related *E. yunnanense* is discussed based on morphological, cytological, and molecular data. It is similar morphologically to *E. yunnanense* but is readily distinguished by having robust (vs. slender), erect (vs. decumbent), and branched (vs. mostly simple), and rather tall stems (60–110 cm vs. 20–60 cm); curved (vs. straight), smooth (vs. torulose), and shorter fruit (5–8 mm vs. 8–15 mm); and fewer ovules per ovary (1–4 vs. 6–10). All examined individuals from different populations of *E. giganteum* clustered into a single clade sister to *E. yunnanense* in phylogenetic analyses using the combined nuclear ITS and plastid DNA datasets. Our cytological studies revealed that the chromosome number of *E. giganteum* is 2*n* = 44, with a genome size of 1160 (±8) Mb, while that of *E. yunnanense* is 2*n* = 28, with a genome size of 718 (±15) Mb. Multiple lines of evidence support the recognition of *E. giganteum* as a distinct species well differentiated from *E. yunnanense*.

## Introduction

*Eutrema* R.Br. (Brassicaceae) is an important genus that includes the model plant for salt-tolerance *E. salsugineum* (Pall.) Al-Shehbaz & Warwick and the economic wasabi plant *E. japonicum* (Miq.) Koidz. This genus was expanded to comprise 26 species (Al-Shehbaz and Warwick 2005; Warwick et al. 2006) with 16 transferred from four previously genera, *Taphrospermum* C.A.Mey., *Thellungiella* O.E.Schulz, *Neomartinella* Pilger, and *Platycraspedum* O.E.Schulz (Al-Shehbaz and Warwick 2005; Warwick et al. 2006). Since then, several new species were described (Ning et al. 2005; Al-Shehbaz 2007; Gan and Li 2014; Xiao et al. 2015; Hao et al. 2015, 2016).

During botanical expeditions to Hengduan Mountains in southwest China from 2013 to 2016, we discovered three populations of *Eutrema* that were strikingly unusual in having large size, big cordate leaves, and stout, erect and branched stems. Only *E. yunnanense* Franch. has such similar big cordate leaves (10–20×10–16 cm), but its stems are slender, decumbent, and rarely branched. Therefore, it was immediately suspected these populations may represent an undescribed new species. In order to further test this hypothesis, morphological, molecular, and cytological studies are presented here on those two species and two related species *E. japonicum* and *E. thibeticum* Franch. were conducted with herein.

## Material and methods

For morphological comparisons and taxonomical treatments, we examined more than ten living individuals from each population of *Eutrema giganteum* (three populations) and *E. yunnanense* (two populations), and photos of all herbarium specimens of *E. yunnanense* preserved in the Chinese Virtual Herbarium (http://www.cvh.org.cn/). We followed Hu et al. (2015) and Hao et al. (2015, 2016) in examining genetic differences between two morphological groups, and three individuals were studied from each population. In order to determine the systematic position of *E. giganteum*, we further sampled two populations each for *E. japonicum* and *E. thibeticum* because they were phylogenetically related to *E. yunnanense*. We sampled one individual each of *E. schulzii* Al-Shehbaz & Warwick, *E. heterophyllum* (W.W. Sm,) H. Hara, *E. verticillatum* (Jeffrey & W.W. Sm.) Al-Shehbaz & Warwick, *E. integrifolium* (DC.) Bunge, *E. altaicum* (C.A. Mey.) Al-Shehbaz & Warwick, and *E. salsugineum* as ingroups. We chose one sample of *Chalcanthus renifolius* (Boiss. & Hohen.) Boiss. as the outgroup. The distribution of sampled populations listed in Table 1. Voucher specimens were deposited in the Sichuan University Herbarium (SZ).

**Table 1.** The sources of materials used for molecular analyses.

Taxon	Voucher	Source	Coordinate	Elevation (m)
*E. giganteum*	J.Quan Liu & G.Q. Hao 15032	Xiling Snow Mountain, Sichuan	30°40'N, 103°09'E	2340
*E. giganteum*	J.Quan Liu & G.Q. Hao 15055	Erlang Mountain, Sichuan	29°50'N, 102°18'E	2480
*E. giganteum*	J.Quan Liu & G.Q. Hao 15069	Gongga Mountain, Sichuan	29°35'N, 102°01'E	2620
*E. yunnanense*	J.Quan Liu & G.Q. Hao 15096	Cangshan Mountain, Yunnan	25°52'N, 99°59'E	3100
*E. yunnanense*	J.Quan Liu & G.Q. Hao 13106	Haba Mountain, Yunnan	25°52'N, 99°59'E	3102
*E. japonicum*	J.Quan Liu & G.Q. Hao 13148	Erlang Mountain, Sichuan	29°51'N, 102°18'E	2300
*E. japonicum*	J.Quan Liu & G.Q. Hao 14001	Longchi, Dujiangyan, Sichuan	31°07'N, 103°48'E	1567
*E. thibeticum*	J.Quan Liu & G.Q. Hao 14003	Jinfo Moutain, Chongqing	28°59'N, 107°11'E	1591
*E. thibeticum*	J.Quan Liu & G.Q. Hao 15031	Xiling Snow Mountain, Sichuan	30°37'N, 103°10'E	1380
*E. integrifolium*	J.Quan Liu & G.Q. Hao 13049	Tian Shan, Xinjiang	43°12'N, 84°49'E	2300
*E. schulzii*	J.Quan Liu & G.Q. Hao 13132	Jianziwan Shan, Sichuan	30°00'N, 100°51'E	4400
*E. salsugineum*		Cultivated, seeds from Shandong		

*All vouchers were housed in the Sichuan University Herbarium (SZ).

We extracted total DNA from silica gel-dried leaves using the modified CTAB method (Doyle and Doyle 1990). The internal transcribed spacer (ITS) and four chloroplast DNA regions (*trn*L-F, *psb*A-*trn*H, *rbc*L, *mat*K) were amplified for phylogenetic analyses. The five pairs of primers used for amplifying and sequencing *trn*L-F, *psb*A-*trn*H, *rbc*L, *mat*K and nuclear nrITS were the same as those used by Hu et al. (2015). PCR amplification and sequencing approaches followed Hu et al. (2015) and Hao et al. (2015). For those ITS sequences with double peaks in those possible hybrids, we further cloned them using vector pGEM-T (Promega, Madison, Wisconsin). We selected ten positive clones for sequencing with primers “sp6” and “t7”. We deposited all new sequences in GenBank under the accession numbers KY969594–KY969625. We aligned DNA sequences using Clustal X (Thompson et al. 1997) and MEGA 5.10 (Tamura et al. 2011) and refined them manually. We concatenated sequences from all four cpDNA fragments into a single matrix for Maximum parsimony (MP) and Maximum likelihood (ML) analyses because of their common inheritance without obvious recombination. To evaluate the congruence of the plastid and nuclear datasets, we employed the incongruence length difference (ILD) test (Farris et al. 1995). The ILD test was carried out using the PAUP* 4.10b (Swofford 2003) with the following settings: 1000 replications, each using a heuristic search with 100 random-addition-sequence replicates and TBR branch swapping. We performed ILD tests between each pair of the cpDNA dataset, and between the nrITS dataset and the combined cpDNA dataset. The *P*-values smaller than 0.01 were considered to be significant incongruent (Cunningham 1997). We reconstructed phylogenetic relationships based on three datasets (nrITS, cpDNAs and combined nrITS+cpDNAs) respectively using MP analyses by PAUP* 4.10b (Swofford 2003), employing a heuristic search with 10,000 replicates and TBR branch swapping. We estimated bootstrap values (Felsenstein 1985) with 1000 replicates and 100 random-addition-sequence replicates per bootstrap replicate. Because indels may contain potential phylogenetic information (Simmons et al. 2001), we coded them using the simple code method by GapCoder (Young and Healy 2003) for phylogenetic analyses. We performed ML analyses using RAxML 7.2.6 (Stamatakis 2006) with the order: raxmlHPC -f a -s sequence. phy -n boot2 -m GTRGAMMA -x 1234 -# 1000 -n outname. We selected the GTRGAMMA model and estimated ML bootstrap analyses with 1000 replicates. We followed Hao et al. (2015) to carry out chromosome number count and genome-size determination.

## Results

### Morphological comparison and geographical distribution

**Figure 1.**
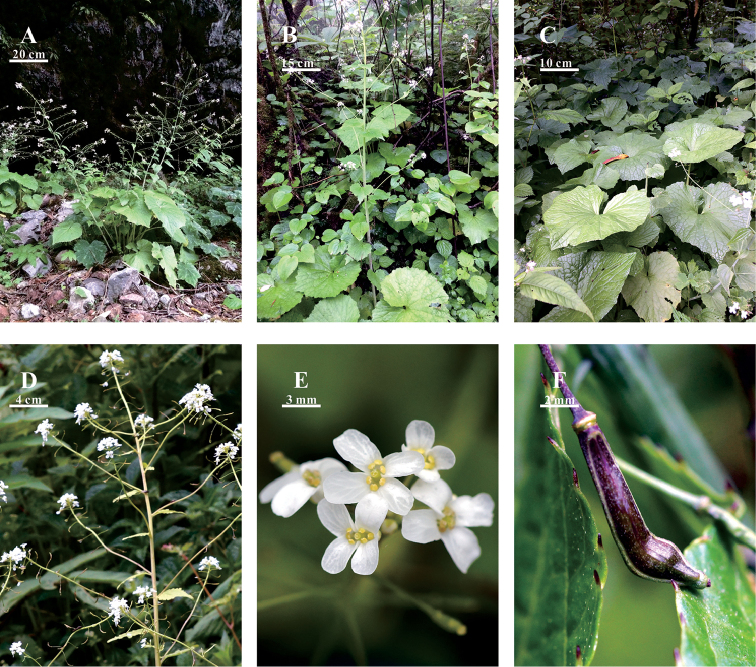
*Eutrema giganteum* G.Q. Hao, Al-Shehbaz & J. Quan Liu. sp. nov. **A–B** Habit **C** Leaves **D** Inflorescence **E** Flowers **F** Fruit.

Our study of herbarium specimens and living plants demonstrated that *Eutrema giganteum* is a morphologically distinctive species. As shown in Fig. 1, it is a glabrous herbaceous perennial, the tallest in the genus, with robust, erect or ascending stems 60–110(–140) cm and alternate branches. Each branch is divaricate-ascending or almost perpendicular to stem and originates from the axil of cauline leaf. The fruits are narrowly oblong, 5–8 × 2–3 mm and curved, but not torulose.

*Eutrema giganteum* is most similar to *E. yunnanense*, but it is readily distinguished from the latter by having stout (vs. slender), erect (vs. decumbent), and branched (vs. rarely branched) stems (60–110 cm vs. 20–60 cm; curved (vs. straight), smooth (vs. torulose), and shorter (5–8 mm vs. 8–15 mm) fruit, and fewer ovules per ovary (1–4 vs. 6–10). Young plants of *E. giganteum* are also somewhat similar to *E. japonicum*. However, they differ in having cordate to reniform (vs. ovate to ovate-cordate) leaf blade. The cultivated plants of *E. japonicum* have stems and fruits similar to those of *E. yunnanense*. The leaves of *E. thibeticum* are similar to those of *E. yunnanense* and *E. giganteum*. However, *E. thibeticum* is comparatively weak and small (20–30 cm tall with basal leaves 2–4 cm).

**Figure 2.**
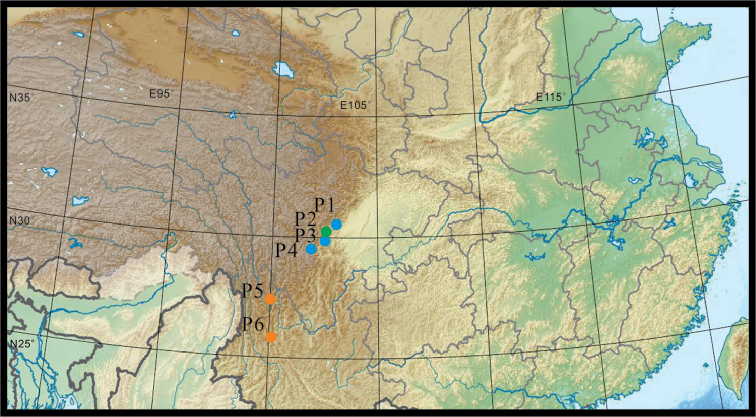
Geographical distribution of *Eutrema giganteum* (P1–P4)and *Eutrema yunnanense* (P5, P6). P1 = Population from Xiling Snow Mountain, Sichuan; P2 = Specimens record from Baoxing County, Sichuan; P3 = Population from Erlang Mountain, Sihuan; P4 = Population from Gongga Mountain, Sichuan; P5 = Population from Haba Mountain, Yunnan; P6 = Population from Cangshan Mountain, Yunnan,

According to specimen records and field investigation, *Eutrema giganteum* is currently known only from Hengduan Mountains in western Sichuan at elevation between 2300 and 2900 m (Fig. 2), while *E. yunnanense* may occurs in the southern part of Hengduan Mountain, Yunnan province, at elevation between 2500 and 3200 m. Zhou et al. (2001) reported that *E. yunnanense* is widely distributed in other provinces of China (for example, Anhui, Gansu, Hebei, Hubei, Hunan, Jiangsu and Zhejiang) at elevation between 400 and 3500 m. Phylogenetic and taxonomic relationships between populations of these provinces and those from Yunnan await future studies.

### Genetic relationship of *Eutrema giganteum* with *E. yunnanense*, *E. japonicum* and *E. thibeticum*

**Figure 3.**
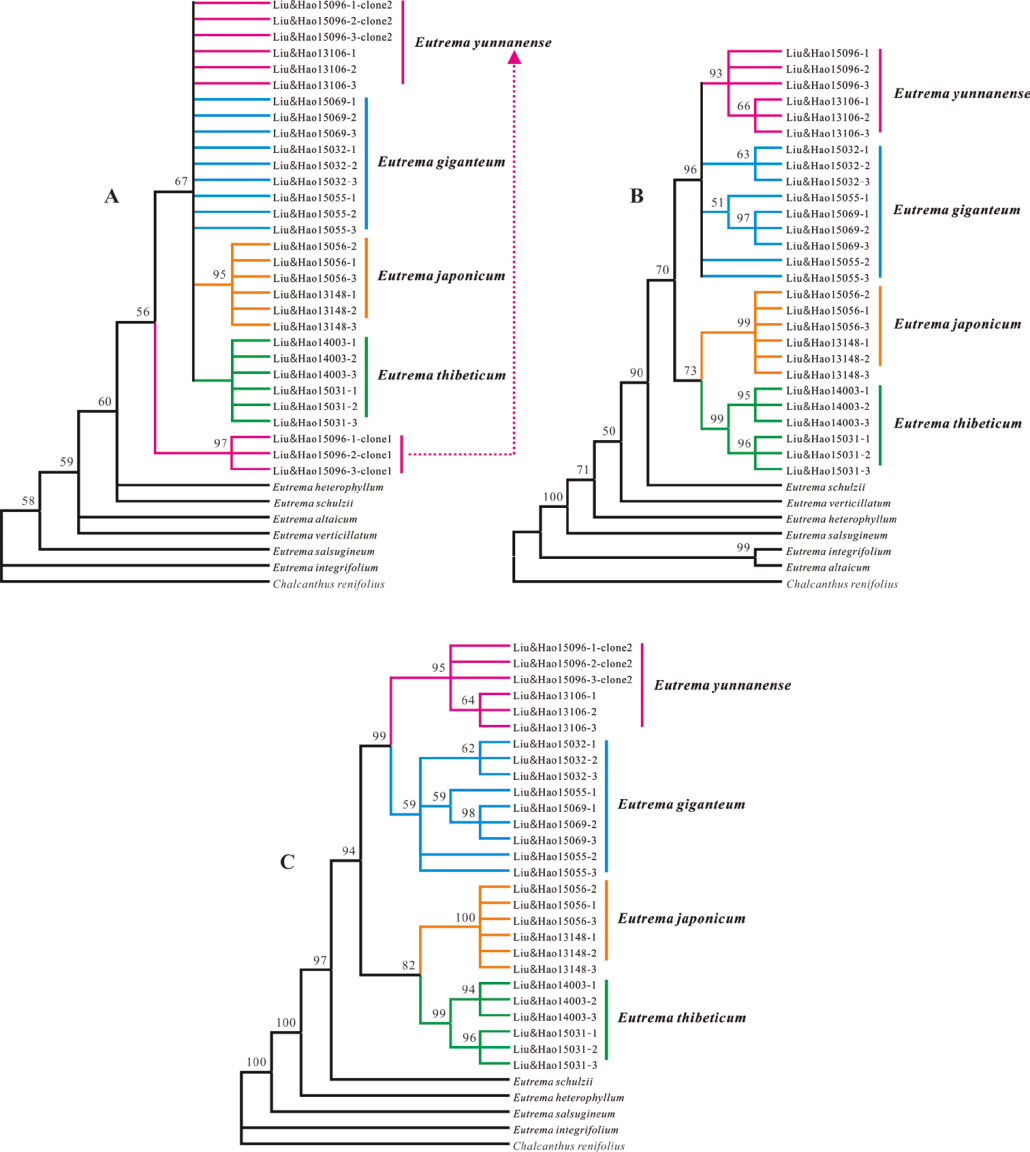
The strict consensus tree constructed based on nrITS data (**A**), four cpDNA regions (**B**) and the combined nrITS and cpDNAs dataset (**C**). Bootstrap support values are given above branches receiving > 50% bootstrap support **A** The 100% strict consensus tree of 667 most maximum parsimony trees based on the analysis of nrITS data **B** The 100% strict consensus tree of 8 trees based on the analysis of 4 cpDNA regions **C** The 100% strict consensus tree of 225 trees based on analysis of combined nrITS and 4 cpDNA regions.

**Figure 4.**
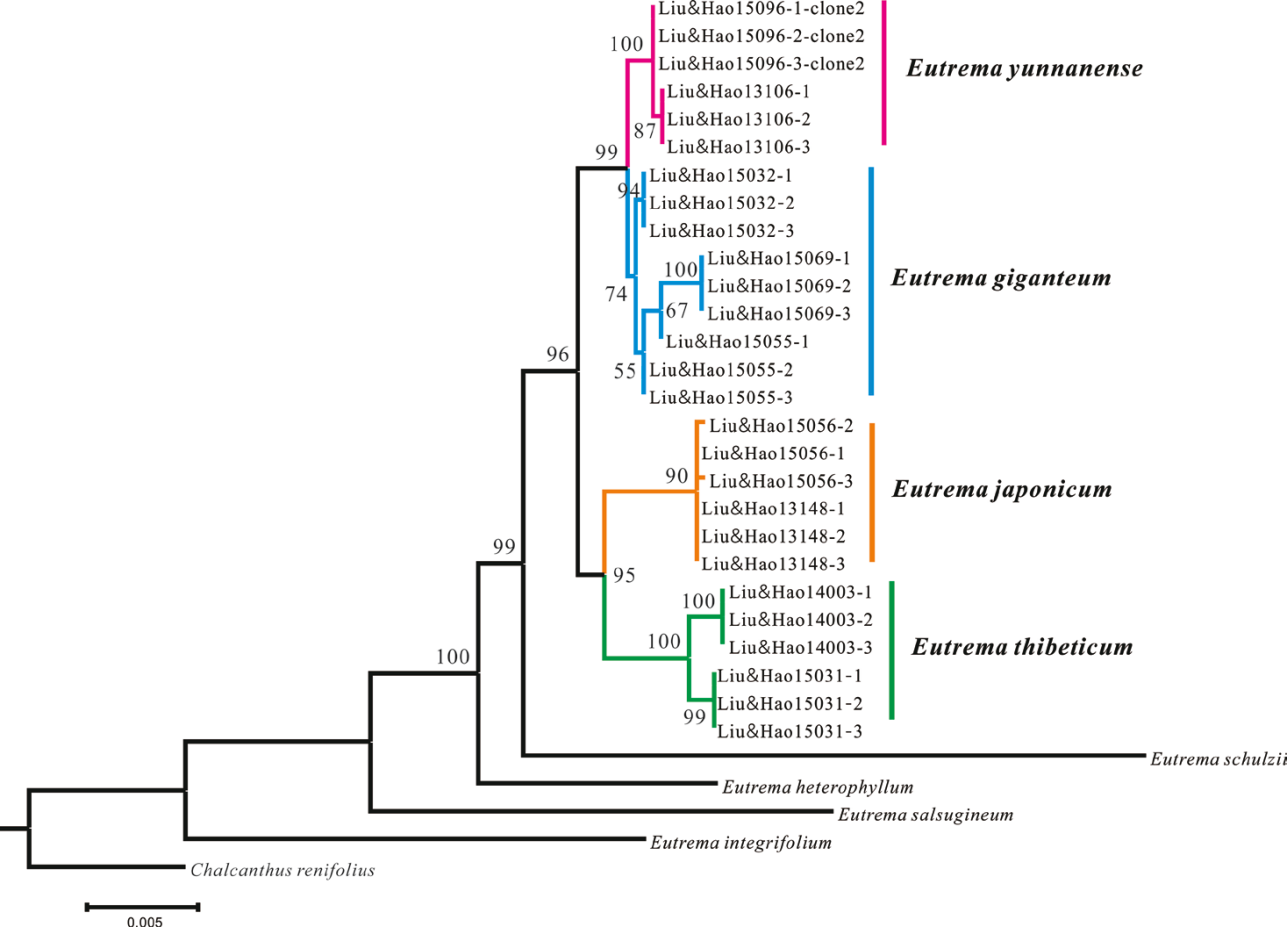
The Maximum likelihood tree based on analysis of the combined nrITS and four cpDNA dataset regions dataset. Bootstrap support values are given above branches receiving > 50% bootstrap support.

Based on sequence variations of nrITS, cpDNAs, and the combined nrITS and cpDNAs (Table 2), phylogenetic analyses suggested that *E. giganteum* is mostly related to *E. yunnanense* (Figs 3, 4). In the MP analyses of nrITS sequence data, *E. giganteum*, one clone of *E. yunnanense*, *E. japonicum*, and *E. thibeticum* formed a single cluster, which together was sister to the other *E. yunnanense* sequences with medium support (50%–70%). In the MP analyses of the sequence variations from cpDNAs, *E. giganteum* and *E. yunnanense* formed a single cluster, then sister to *E. japonicum* and *E. thibeticum* with higher support (>70%). The *P*-values resulting from the ILD tests show that there is significant incongruence between the four cpDNA and nrITS when including all species (*P* = 0.003). After removing conflicting sequences of *E. yunnanense*, *E. altaicum* and *E. verticillatum*, the *P* value rose to 1.000, indicating that there is no significant incongruence between nrDNA and cpDNA datasets. We therefore combined them for further analyses. In the MP analyses of the combined dataset, all *E. giganteum* individuals formed a single cluster, sister to the cluster comprising *E. yunnanense* individuals with medium supports (50%–90%). The clade comprising both of them was sister to the clade formed by both *E. japonicum* and *E. thibeticum* with a high support (94%) (Fig. 3). ML analyses produced similar tree topologies to MP trees but the supports were higher than MP analyses (Fig. 4).

**Table 2.** Tree statistics for analyses of the seven data sets.

**Data set**	ITS^*^	***psb*A-*trn*H**	***trn*L-F**	***rbc*L**	***mat*K**	Combined cpDNA^*^	Combined cpDNA and ITS^*^
No. of sequences	37^**^	34	34	34	34	34	32^**^
Aligned length used in analyses	606	332	665	482	728	2288	2894
No. of variable characters	97	64	54	7	61	238	280
No. of parsimony-informative characters	34	20	26	4	23	93	96
Tree length (steps)	138	78	59	7	68	291	330
Consistency (CI)	0.804348	0.910256	0.983051	1	0.911765	0.859107	0.900000
Retention index (RI)	0.795455	0.907895	0.986301	1	0.934783	0.880117	0.909341
Rescaled consistency index (RC)	0.639822	0.826417	0.969584	1	0.852302	0.756114	0.818407

*gaps were coded and included;

**the cloned sequences were included

### Chromosome number and genome size

**Figure 5.**
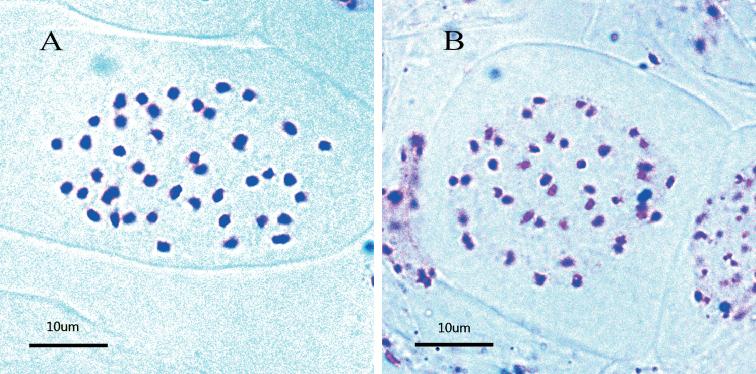
Photomicrographs of mitotic chromosomes of *Eutrema giganteum*. **A** population from Xiling Snow Mountain **B** population from Erlang Mountain.

Two populations of *Eutrema giganteum* from Xiling Snow Mountain and Erlang Mountain, and one population of *E. yunnanense* from the type locality, Cangshan Mountain, were cytologically examined. Mitotic chromosome number of *E. giganteum* was determined as 2*n* = 44 (Fig. 5), while that of *E. yunnanense* was 2*n* = 28, as the same as counted by Du and Gu (2004). Genome size of *E. giganteum* was determined as 1160 (±8) Mb while that of *E. yunnanense* was 718 (±15) Mb.

## Discussion

Different species concepts emphasize the different criteria to define a new species (Wheeler and Meier 2000). An integrative practice using multiple criteria to circumscribe species boundaries and define a new species will produce relatively objective and operational taxonomy (Su et al. 2015; Hu et al. 2015; Liu 2016). Based on morphological, cytological, and molecular data, the new species *E. giganteum* is described here as a new species distinct from the closely related *E. yunnanense*. First, our observations of herbarium specimens and living plants suggested that *E. giganteum* is most similar to *E. yunnanense*, but could be distinguished by distinct morphological traits, including stem, fruit, ovule number, and individual size. Second, a species should be delimited as an evolutionarily distinct lineage (de Queiroz 1998, 2007; Stockman and Bond 2007; Fujita et al. 2012; Hendrixson et al. 2013; Mckay et al. 2013). Our molecular phylogenetic analyses of combined nrITS and cpDNA datasets, all examined individuals of *E. giganteum* clustered into a single lineage, sister to *Eutrema yunnanense*. Third, chromosome number count and genome-size measure revealed the obviously cytological discrepancy between *E. giganteum* and *E. yunnanense*. The chromosome number of *E. giganteum* is 2*n* = 44, whereas *E. yunnanense* has the number of 2*n* = 28. Consistent with the difference of the chromosome number, we also found that the genome size of *E. giganteum* is almost 1.5 times larger than that of *E. yunnanense*. These chromosomal and genomic differences are likely to lead to the obvious reproductive isolations between *E. giganteum* and *E. yunnanense*. In addition, our unpublished data suggested that some populations of *E. thibeticum* have the chromosome number of 2*n* = 16. Both *E. giganteum* and *E. thibeticum* are co-distributed in the Hengduan Mountains in western Sichuan where the former occurs at the high elevation while the latter at the low elevation. It seems likely that *E. giganteum* (2*n* = 44) originated from a hybridization between *E. thibeticum* (2*n* = 16) and *E. yunnanense* (2*n* = 28) although further molecular evidence and experimental hybridization are needed. Overall, all available lines of evidence suggest that *E. giganteum* should be recognized as a distinct new species.

## Taxonomic treatment

### Eutrema giganteum

Taxon classificationPlantaeBrassicalesBrassicaceae

G.Q. Hao, Al-Shehbaz & J. Quan Liu sp. nov.

urn:lsid:ipni.org:names:77163811-1

#### Type.

China. Sichuan: Dayi County, Xiling Snow Mountain, Heishuihe Giant Panda Nature Reserve, 30°40'22"N, 103°9'47"E, 2340 m, 6 July 2015, *J.Quan Liu & G. Q. Hao 2015032-1* (Holotype, SZ)., *J.Quan Liu & G. Q. Hao 2015032-2* (Isotype, SZ), *J.Quan Liu & G. Q. Hao 2015032-3* (Isotype, SZ). Figure 1.

#### Etymology.

The specific epithet refers to the remarkably huge plant size. The erect stem can extend to around 60–110 (–140) cm, higher than all the other *Eutrema* species.

#### Description.

Herbs, perennial, glabrous or sparsely pilose on upper parts; rhizome fleshy, to 2 cm in diam. Stems 60–110(–140) cm tall, robust, to ca. 1 cm diam, erect or ascending, simple at base, alternately branched above; branches 1-leaved, divaricate-ascending or almost perpendicular to stem. Basal leaves rosulate; petiole with a groove, hollow, cylindrical, (12–)15–26 (–35) cm; leaf blade cordate, (18–)25–35(–40) × (15–)20–30(–35) cm, margin dentate, denticulate or repand, with distinct apiculate callosities terminating ultimate veins, apex subacute to acuminate; cauline leaves with petioles gradually shorter upward, cordate to lanceolate, lowermost cauline 6–10 × 3–7 cm, gradually reduced in size upward. Racemes ebracteate, lax, elongated in fruit, main branch 20–30 cm; fruiting pedicels slender, reflexed or spreading, 0.6–1.5(–2.2) cm. Sepals ovate or oblong, 1.5–2 × ca. 1 mm, deciduous; petals white, oblong-spatulate, 3.5–5(–7) × 1.5–2 mm, apex obtuse to rounded; claw present; filaments white, 3–4.5 mm; ovules 1–4 per ovary. Fruit dehiscent silicles, narrowly oblong, 5–8 × 2–3 mm, curved, not torulose; valves with an obscure midvein; gynophore absent or obsolete, septum complete. Seeds oblong, 2.5–3.5 × 1.5–2.0 mm.

#### Phenology.

Flowering: April–July; fruiting: May–August.

#### Distribution and habitat.

*Eutrema giganteum* is currently known from Hengduan Mountains in western Sichuan, China, including Xiling Snow Mountain, Jiajin Mountain, Erlang Mountain, and Gongga Mountain (Fig. 2). It grows in shady, humid forests at elevation of 2200–2900 m.

#### Additional specimens examined

**(paratype).** China: Sichuan: Baoxing County, 1954, *Z. P. Song 38379* (KUN); Baoxing County, Puxi Gou, April 1959, 2700 m, *Sichuan Economic Plant Investigation Team 00324* (CDBI); Luding County, Dawanzi, 2300 m, 2 May 1980, *Q. Q. Wang 22061* (CDBI); Dayi County, Heishuihe Nature Reserve, 2900 m, 6 June 2007, *D. H. Zhu, Z. B. Feng, C. Zhang & F. Wang 20070659* (PE).

## Supplementary Material

XML Treatment for Eutrema giganteum
